# Decreased Frequency of Intestinal Regulatory CD5^+^ B Cells in Colonic Inflammation

**DOI:** 10.1371/journal.pone.0146191

**Published:** 2016-01-04

**Authors:** Yoshiyuki Mishima, Shunji Ishihara, Akihiko Oka, Nobuhiko Fukuba, Naoki Oshima, Hiroki Sonoyama, Noritsugu Yamashita, Yasumasa Tada, Ryusaku Kusunoki, Ichiro Moriyama, Takafumi Yuki, Kousaku Kawashima, Yoshikazu Kinoshita

**Affiliations:** 1 Department of Internal Medicine II, Shimane University Faculty of Medicine, Izumo, Shimane, Japan; 2 Cancer Center, Shimane University Hospital, Izumo, Shimane, Japan; Massachusetts General Hospital, UNITED STATES

## Abstract

**Background:**

CD5^+^ B cells are a type of regulatory immune cells, though the involvement of this B cell subset in intestinal inflammation and immune regulation is not fully understood.

**Methods:**

We examined the distribution of CD5^+^ B cells in various mouse organs. Expression levels of CD11b, IgM, and toll-like receptor (TLR)-4 and -9 in B cells were evaluated. *In vitro*, TLR-stimulated IL-10 production by colonic lamina propria (LP) CD5^+^ and CD5^-^ B cells was measured. *In vivo*, mice with acute or chronic dextran sulfate sodium (DSS)-induced colonic injury were examined, and the frequency of colonic LP CD5^+^ B cells in those was assessed by flow cytometry.

**Results:**

The expression level of TLR9 was higher in colonic LP CD5^+^ B cells as compared to CD5^-^ B cells. Colonic LP CD5^+^ B cells produced greater amounts of IL-10 following stimulation with TLR ligands, especially TLR9, as compared with the LP CD5^-^ B cells. Acute intestinal inflammation transiently decreased the frequency of colonic LP CD5^+^ B cells, while chronic inflammation induced a persistent decrease in colonic LP CD5^+^ B cells and led to a CD5^-^ B cell-dominant condition.

**Conclusion:**

A persistent altered mucosal B cell population caused by chronic gut inflammation may be involved in the pathogenesis of inflammatory bowel diseases.

## Introduction

B cells are generally well known as antibody-producing cells that play a key role in adaptive immune response under physiological and pathological conditions.[1] However, their role in innate immune-related disorders has not been fully elucidated. CD5^+^ B cells are a unique subset of B cells localized mainly in peritoneal and pleural cavities, and appear to be involved in an innate immune system that is able to sense pathogen-associated molecular patterns (PAMPs) and initiate immune response by secretion of natural polyreactive antibodies,[2] thus limiting bacterial spread before induction of an adaptive immune reaction. It has been shown that CD5^+^ B cells have protective roles against influenza and schistosomal infection through natural IgM antibodies or Fas ligands.[3,4] In contrast, CD5^-^ B cells, predominately localized in the spleen and lymph nodes, participate in adaptive immunity by producing antigen-specific antibodies that interact with helper T cells.

Previous studies have reported conflicting results regarding the functions of CD5^+^ B cells in autoimmune diseases. Autoantibodies produced by those cells have been identified as potentially contributing to the development of systemic lupus erythematosus (SLE), rheumatoid arthritis (RA), and Sjogren’s syndrome,[5–7] and depletion of this subset decreases pathogenic autoantibodies, leading to a reduction of disease activity. On the other hand, CD5^+^ B cells are associated with regulation of experimental autoimmune encephalitis (EAE) through secretion of anti-inflammatory cytokines or decreased expression of the chemokine receptor CXCR5.[8,9] Therefore, CD5^+^B cells may function as a double-edged sword in autoimmune and infectious diseases, though the discrepant findings thus far presented might be related to the timing of intervention with this B cell subset.

Of note, multiple studies have demonstrated that CD5^+^ B cells are protective in cases of intestinal inflammation by generating natural antibodies against microbial flora[10] and producing IL-10.[11,12] Intestinal CD5^+^ B cells are also the main source of IgA secretion,[13,14] which can continuously protect the body from threat of infection by pathological bacteria and maintain a normal flora composition. Therefore, intestinal CD5^+^ B cells appear to have important roles in regulatory immunity in cases of mucosal inflammation and homeostasis.

In the present study, we investigated the involvement of colonic CD5^+^ B cells and compared them with CD5^-^ B cells using model mice with acute or chronic colitis. Our results demonstrate that chronic intestinal inflammation decreases the frequency of CD5^+^ B cells and leads to a sustained CD5^-^ B cell-dominant condition, which may be involved in the pathogenesis of refractory inflammatory bowel diseases (IBD).

## Materials and Methods

### Reagents

The following reagents were employed. For flow cytometry: FITC-, PE-, and PE-Cy5-conjugated anti-mouse CD5 (53–7.3), B220 (RA3-6B2), CD11b (M1/70), IgM (R6-60.2) (BD Biosciences-Pharmingen, San Jose, CA), TLR4/MD2 (UT41), and TLR9 (Imgenex Biotech, Orissa, India). For B cell isolation: FcR blocking reagent, anti-CD5 microbeads (catalog # 130-049-301) and a B cell isolation kit, mouse (catalog # 130-090-862) (Miltenyi, San Diego, CA). For cell cultures: ultra-pure *E*.*coli* LPS (0111:B4 strain) (Invivogen, San Diego, CA). Unmethylated CpG-DNA (5'-TGACTGTGAACGTTCGAGATGA-3') was synthesized by Hokkaido System Science Co., Ltd. (Hokkaido, Japan).

### Animals and experimental colitis

Seven-week-old male specific pathogen-free BALB/c mice were obtained from Nihon Clea (Tokyo, Japan). After an initial adaptation period of 1 week, a dextran sulfate sodium (DSS) (5 kDa; Wako Pure Chemical Industries, Osaka, Japan) solution (2.5% w/v) was administered to the experimental group as drinking water for 7 days, with the solution changed every other day, to induce colitis. Mouse conditions were carefully monitored three times a week after DSS administration until termination of the study. The control group was given normal water lacking DSS. In parallel, chronic colitis was induced in some mice by repeating administrations of DSS solution. Each cycle consisted of a 7-day exposure to DSS, followed by a 14-day period without DSS, which continued for up to 7 cycles. At 14 days after the DSS period following completion of 1, 3, 5, or 7 cycles, mice were euthanized under diethyl ether anesthesia by quick cervical distortion to minimize animal suffering and evaluated. Two mice in the chronic colitis model group were excluded from analysis, because of development of colorectal tumors (after 5 and 7 cycles, respectively). This study was carried out in strict accordance with the recommendations in the Guide for the Care and Use of Laboratory Animals of the National Institutes of Health. The protocol was approved by the Institute for Animal Experimentation of Shimane University (Protocol Number: IZ21-108).

### Cell isolation

Mononuclear cells were isolated from several of the mouse organs, using a method previously described.[15] Peritoneal cavity (PerC) cells were collected after intraperitoneal injection of Ca^2+^- and Mg^2+^-free Hanks' balanced salt solution (HBSS; Gibco-Invitrogen) with 2% fetal bovine serum (FBS; ICN Biomedicals, Aurora, OH, USA). Mesenteric lymph nodes (MLN) were crushed through 70-μm filters into phosphate-buffered saline (PBS) with 2% FBS. Spleens were mechanically dissociated and red blood cells were lysed in ammonium phosphate/chloride lysis buffer. For isolating colon lamina propria mononuclear cells (LPMC), we used only the distal part of the colon, which is the area susceptible to DSS-induced colitis. Colons were opened longitudinally and washed extensively with cold PBS, then cut into 5-mm pieces. Obtained tissues were incubated in 1 mM DTT (Sigma-Aldrich, St. Louis, Missouri, USA) for 15 minutes at room temperature and then 1 mM EDTA 3 times at 37°C for 20 minutes each, followed by HBSS with 1 mg/ml of collagenase type 3 (Worthington Biochemical Corporation, Lakewood, New Jersey, USA), 0.1 mg/ml of DNase I (Worthington Biochemical Corporation), 2% FBS, and 1% penicillin-streptomycin (Gibco-Invitrogen) for 60 minutes at 37°C. Cell suspensions were filtered through a nylon mesh and centrifuged, then LPMC were purified using a 44–70% discontinuous Percoll gradient (GE Healthcare, Buckinghamshire, UK). After centrifugation at 800 x *g* for 20 minutes at 22°C, cells were collected from the interface, and washed and re-suspended in PBS with 2% FBS. Cell viability was greater than 90%, as determined by eosin Y exclusion.

### Colonic LP CD5^+^ and CD5^-^B cell purification, and cell cultures

To evaluate TLR-mediated IL-10 secretion by LP CD5^+^ and CD5^-^ B cells, colonic LPMC were incubated with an FcR blocking reagent on ice for 10 minutes, then B cells were isolated by negative selection with a B cell-isolation kit magnetically. The negative fractions (whole B cells) were further purified using anti-CD5 microbeads for CD5^+^ and CD5^-^ B cells. All selections were performed according to the manufacturer’s instructions. Final CD5^+^ and CD5^-^ B cell fractions were confirmed to be greater than 81% and 83% pure, respectively, using flow cytometry. Colonic LP CD5^+^ and CD5^-^ B cells (5 x 10^5^) were separately cultured at 200 μl/well in 96-well plates for 72 hours at 37°C with 5% CO_2_. The culture medium was RPMI 1640 (Gibco-Invitrogen) containing 10% FBS and 1% penicillin-streptomycin-amphotericin B (Gibco-Invitrogen), with or without LPS (100 ng/ml) or CpG-DNA (1 nM). Following the cell cultures, the supernatants were collected for measurements of IL-10 by ELISA.

### Flow cytometry

Three-color flow cytometric analyses were performed. Cells were stained with appropriate antibodies on ice, as previously described, for 20 minutes to detect cell surface markers. In some experiments, cells were further fixed and permeabilized with Intraprep (Beckman Coulter, Fullerton, CA, USA) and stained intracellularly with anti-TLR9. After washing, the cells were immediately subjected to flow cytometry (EPICS XL, Beckman Coulter, Tokyo, Japan), and analyzed using EXPO32^TM^ software. Isotype-matched antibodies were used to determine the level of non-specific staining.

### Measurement of IL-10 level

The level of IL-10 in supernatant was measured using a mouse IL-10 Quantikine ELISA Kit (R&D systems, Minneapolis, MN), according to the manufacturer’s instructions. Briefly, appropriate sample amounts were transferred into the wells of anti-mouse IL-10-coated micro-titer strips and a secondary biotinylated monoclonal antibody was added. After washing, the samples were incubated with streptavidin-peroxidase. A substrate solution was then added to produce color directly proportional to the concentration of IL-10 present in the sample. Quantitative results were obtained from a standard curve produced from the experimental findings. IL-10 levels in the TLR-stimulated cultures were normalized by that in the cultures without TLR ligands.

### Statistical analysis

All data are expressed as the mean ± standard deviation (SD). Values were analyzed using Student’s t test and Spearman’s rank correlation with Stat-View 4.0 software (Abacus Concepts, Inc., USA). For comparisons of multiple values, ANOVA was used. P values less than 0.05 were considered to be significant.

## Results

### Distribution and characteristics of CD5^+^ and CD5^-^ B cells in normal mice

**Fig 1:**
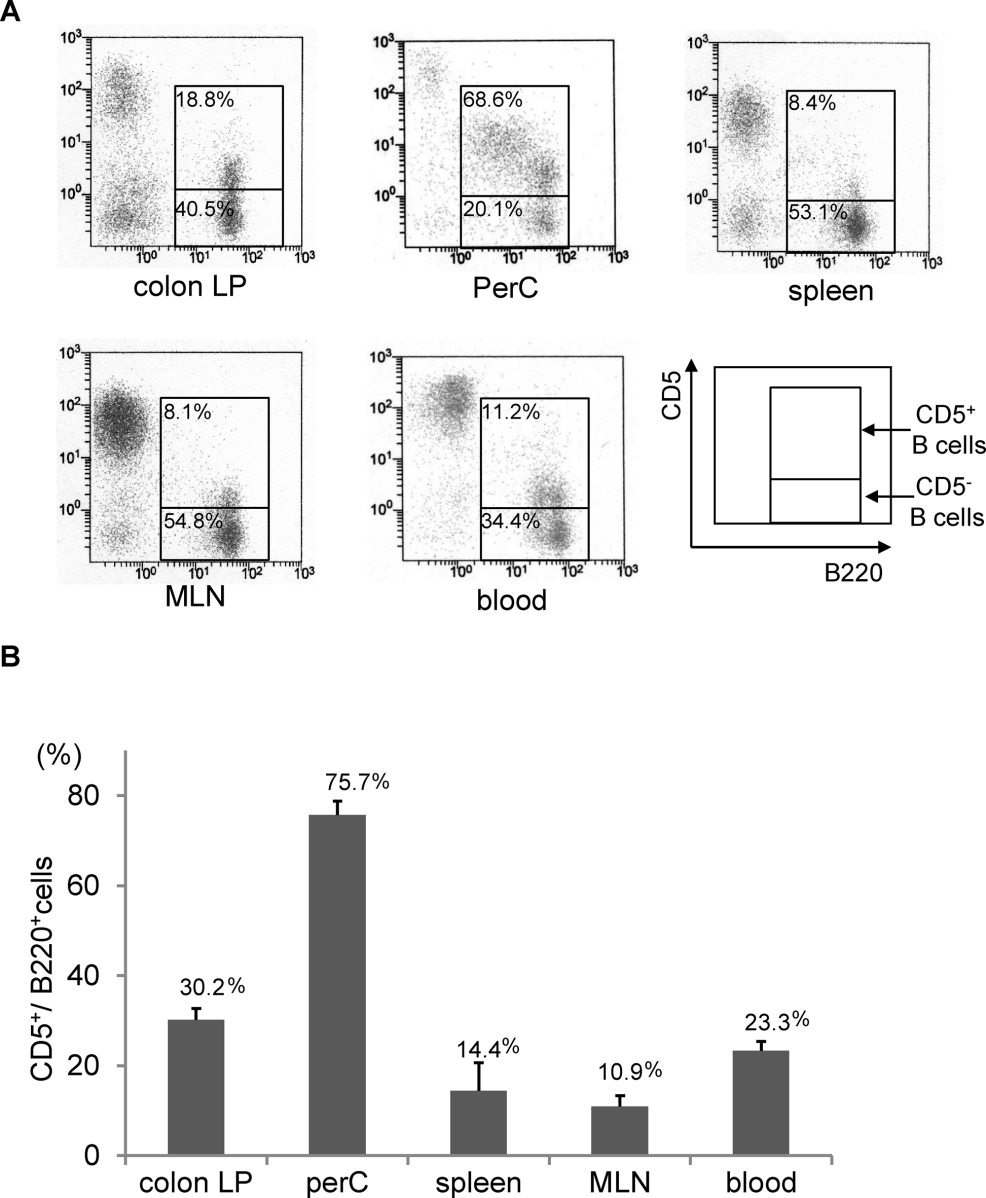
Distribution and characteristics of CD5^+^ and CD5^-^ B cells in normal mice. Mononuclear cells were isolated from peritoneal cavity (PerC), mesenteric lymph nodes (MLN), colon lamina propria (LP), spleen, and blood samples, then stained with anti-B220 and CD5 antibodies, and analyzed by flow cytometry. **(A)** Representative images. **(B)** Frequency of CD5^+^ B cells appearing in B220^+^ B cell population. N = 5, performed in triplicate. Data are presented as the mean ± SD.

We first investigated the distribution of CD5^+^ and CD5^-^ B cells in normal mice. Colonic LP CD5^+^ B cells comprised approximately 30% of the total LP B cell population with a high intensity of B220 and low level of CD5 (B220^high^CD5^low^), similar to MLN, peripheral blood, and splenic CD5^+^ B cells. On the other hand, CD5^+^ B cells among PerC comprised 75.7% of the total B cell population, though there seemed to be two different populations of those cells in PerC (B220^mid^CD5^mid^ and B220^hi^CD5^lo^) **(Fig 1A and 1B)**. These results indicate that the majority of intestine-related (MLN, colonic LP) B cells are generally included in the CD5^-^ conventional B cell subset (B-2 cells), while the major population of PerC B cells is the CD5^+^ B cell subset (B-1a cells), which are functionally different from B-2 lineage cells.

**Fig 2:**
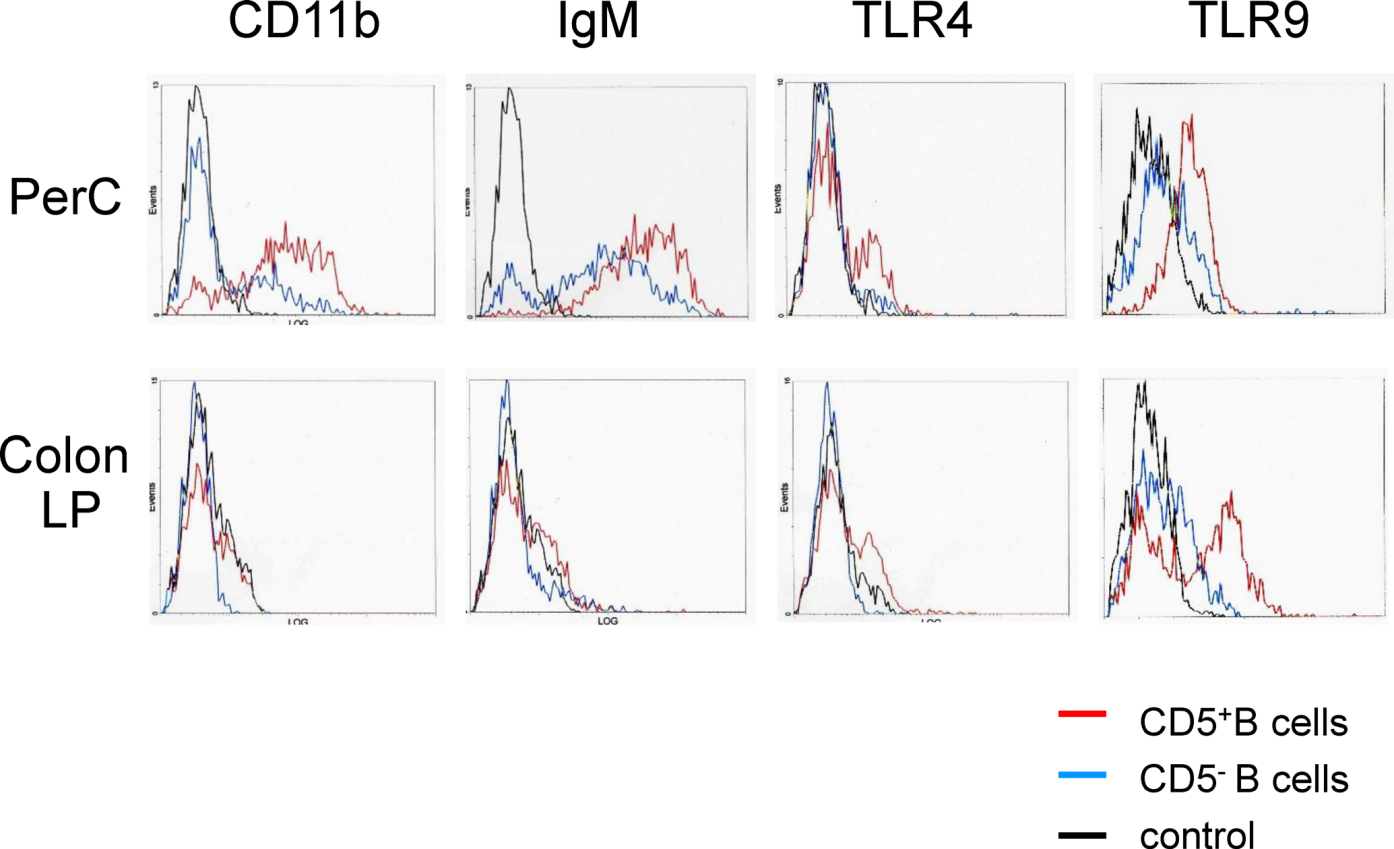
Immunological characteristics of intestinal CD5^+^ B cells differ from those of peritoneal CD5^+^ B cells. The expression levels of anti-CD11b, IgM, and TLR4/MD2 on CD5^+^ and CD5^-^ B cells obtained from the colonic LP or peritoneal cavity (PerC) of normal mice were evaluated by flow cytometry. In parallel, B cells were intracellularly stained with the anti-TLR9 antibody after the cell surface staining with anti-B220 and CD5 antibodies, and examined using flow cytometry. N = 3, performed twice.

Next, we characterized the B cell subsets by assessing the cell surface antigens IgM and CD11b as B-1 cell-related markers, in addition to TLRs in CD5^+^ and CD5^-^ B cells. Although the expression levels of CD11b, IgM, and TLR4 in PerC CD5^+^ B cells were not greatly different from those in PerC CD5^-^ B cells, the former expressed a higher level of TLR9. Colonic LP CD5^+^ B cells possessed IgM^mid^ CD11b^low^ **(Fig 2)**, which was a similar expression pattern as seen with spleen, MLN, and peripheral blood CD5^+^ B cells **(data not shown)**. On the other hand, colonic LP CD5^***-***^ B cells generally show weak expression of these cell surface antigens (IgM^low^ CD11b^-^) as compared with colonic LP CD5^***+***^ B cells. Of note, colonic LP CD5^+^ B cells showed greater levels of TLR9 but not TLR4 as compared to either colonic LP CD5^-^ B cells or PerC CD5^+^ B cells **(Fig 2)**. Taken together, the population and phenotype of CD5^***+***^ B cells in the colonic LP were different from those of PerC CD5^***+***^ B cells

### Intestinal CD5^+^ B cells produce a greater amount of IL-10 in response to TLR stimulation than CD5^-^ B cells

**Fig 3:**
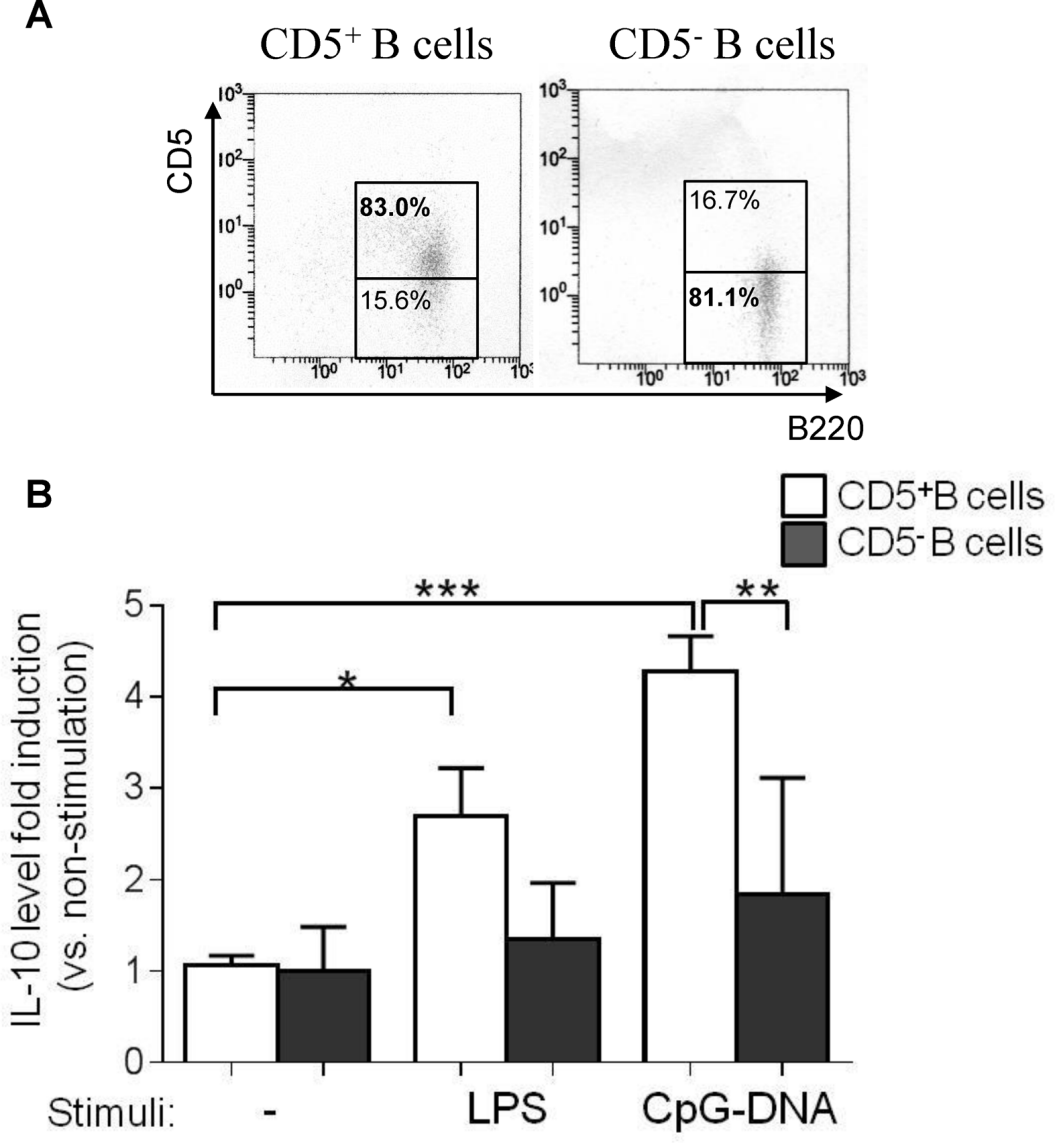
Intestinal CD5^+^ B cells produce greater level of IL-10 than CD5^-^ B cells in response to Toll-like receptor ligands. **(A, B)** Normal mouse purified CD5^+^ and CD5^-^ colonic LP B cells (5x10^5^) were separately cultured with or without 100 ng/ml LPS or 1 nM CpG-DNA for 3 days. The concentration of IL-10 in the supernatants was assessed by ELISA. The IL-10 level in cultures with TLR stimulation was normalized by that in cultures without TLR stimulation. The experiment was performed in triplicate. Data are presented as the mean ± SD. *p<0.05, **p<0.01, ***p<0.001

Since splenic and PerC CD5^+^ B cells are protective against colitis,[10,12] we examined the relative production levels of the anti-inflammatory cytokine IL-10 in TLR-stimulated colonic LP CD5^+^ and CD5^-^ B cells *in vitro* **(Fig 3A)**. Colonic LP CD5^+^ B cells produced greater amounts of IL-10 following stimulation with TLR ligands, especially TLR9, as compared with the LP CD5^-^ B cells **(Fig 3B)**, which might have been due to the higher expression level of TLR9 in intestinal CD5^+^ B cells **(Fig 2)**. These results suggest that TLR9-medeated IL-10 production may be one of the regulatory mechanisms of intestinal CD5^+^ B cells in regard to gut inflammation and homeostasis.

### Acute intestinal inflammation transiently decreases the frequency of mucosal CD5^+^ B cells

Although B cells attenuate colitis through multiple mechanisms,[12,16,17] little is known regarding whether intestinal inflammation itself influences the involvement of B cell subsets. Therefore, we investigated the frequency of the colonic LP B cell subpopulation in response to intestinal inflammation.

**Fig 4:**
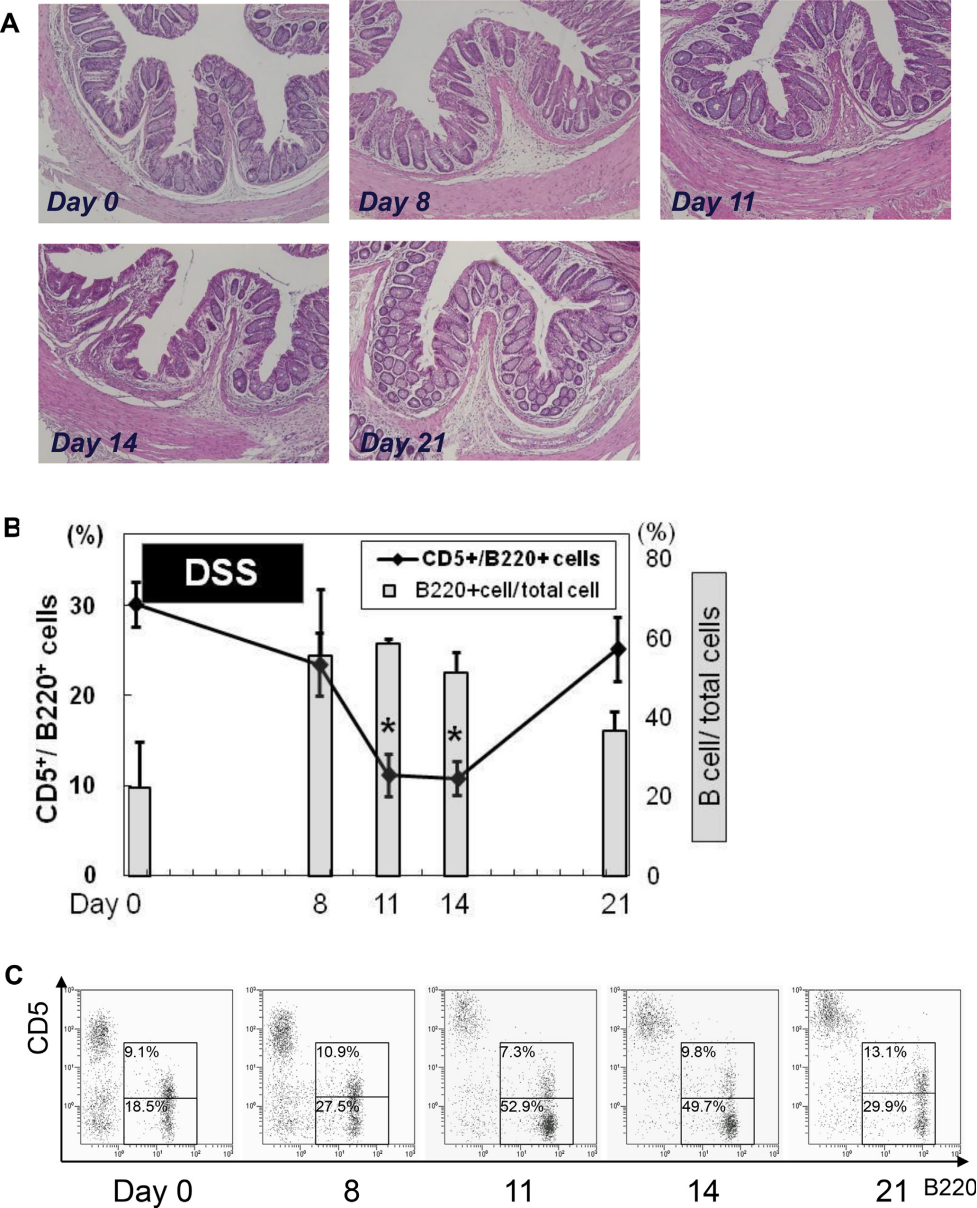
Acute intestinal inflammation transiently decreases frequency of colonic LP CD5^+^ B cells. Mice were administered a 2.5% DSS solution for 7 days and colonic tissues were harvested on days 8, 11, 14, and 21. **(A)** Representative histological images. **(B)** The relative percentage of B cells among colonic LPMC (gray histogram) and ratio of CD5^***+***^ B cells among total B cells (solid line) were assessed using flow cytometry. **(C)** Representative flow cytometry dot blot findings. N = 4–5 at each time point. Data are presented as the mean ± SD. *p<0.05.

Using an acute model of DSS-induced colitis, histological inflammation characterized by massive cellular infiltration with edema was shown on day 8, and then the severity of colitis decreased and nearly recovered morphologically by day 21 **(Fig 4A)**. Interestingly, the population of colon LP CD5^***+***^ B cells among total B cells (30.2±2.5%) was significantly decreased on day 8 (23.5±3.5%) and minimal on day 14 (10.8±1.9%, 35.7% decrease as compared with non-inflamed colon). Thereafter, the population began to restore and finally recovered up to 25.2±3.5% (83.4% decrease as compared with control) on day 21 **(Fig 4B and 4C)**. The ratio of CD5^+^ B cells among PerC was not significantly affected by mucosal inflammation **(S1A Fig)**

### Chronic intestinal inflammation persistently decreases frequency of CD5^+^ B cells

Although acute mucosal inflammation transiently alters the distribution of colonic LP B cells, human IBD is associated with chronic intestinal inflammation with repeating relapse and remission. Therefore, we next examined the intestinal B cell subsets in a chronic mucosal injury model.

**Fig 5:**
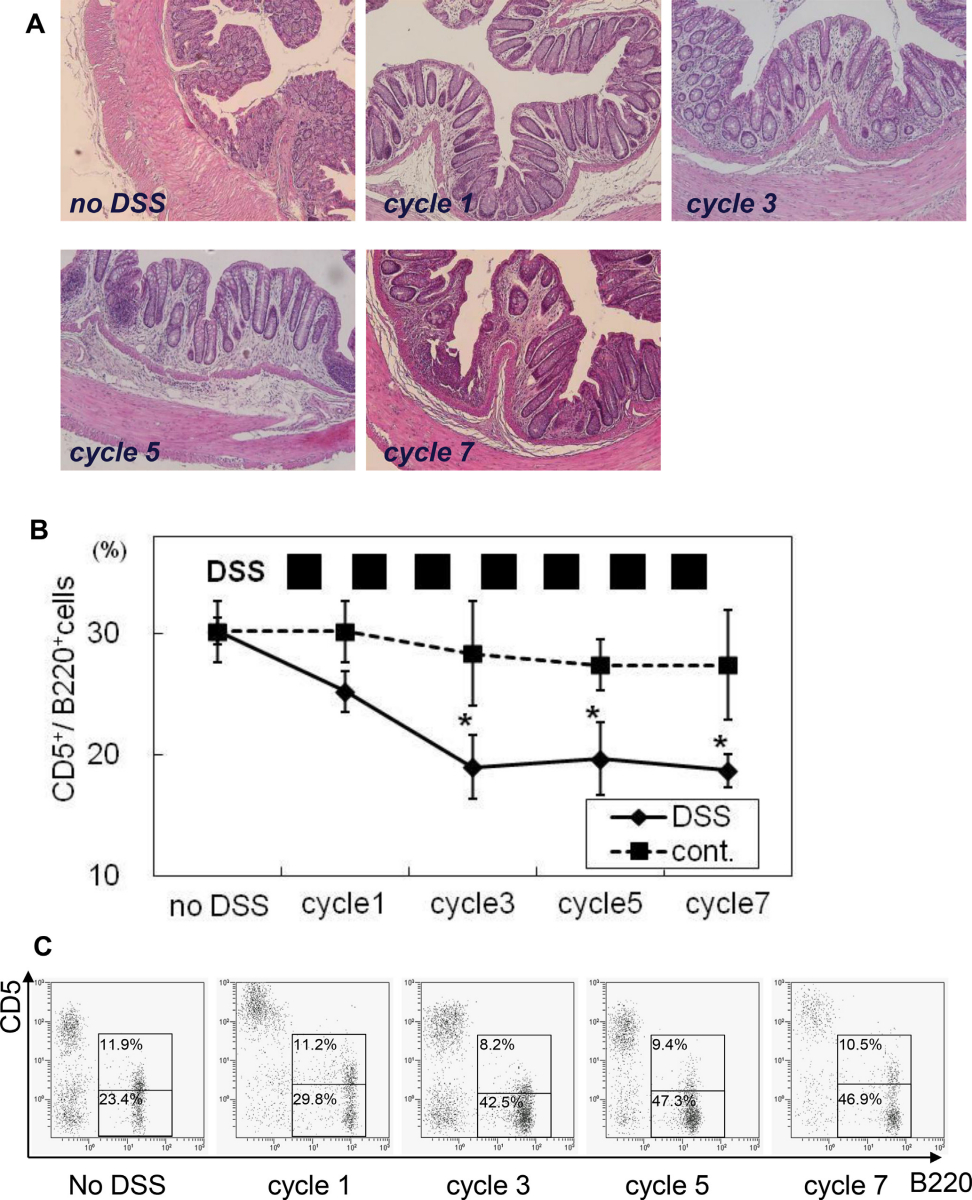
Chronic intestinal inflammation persistently decreases frequency of mucosal CD5^+^ B cells. Chronic colitis was induced by repeated administrations of DSS solution. Each cycle consisted of a period of 7 days with DSS, followed by 14 days without DSS, which continued for up to 7 cycles. Mice were euthanized on day 21 of cycles 1, 3, 5, and 7. **(A)** Distal colon portions with or without DSS treatment were histologically evaluated. The numbers of CD5^+^ B cells among **(B)** colonic LP cells were determined using flow cytometry. **(C)** Representative flow cytometry dot blot findings. The experiments were repeated in triplicate. N = 6 at each time point. Data are presented as the mean ± SD. *p<0.05.

Although the 14-day recovery period after ending DSS treatment (day 21) was nearly enough to recover histological inflammation and alternation of B cell subsets in acute colitis, repeated DSS treatments resulted in development of chronic intestinal inflammation characterized by thickened mucosal and submucosal layers, moderate mononuclear-cell filtration, and huge lymphoid aggregations on day 21 during the third, fifth, and seventh cycles **(Fig 5A)**. Notably, chronic intestinal inflammation significantly decreased the frequency of CD5^***+***^ B cells over time, resulting in a sustained CD5^-^ B cell-dominant condition **(Fig 5B and 5C)**, whereas the ratio of CD5^+^ and CD5^-^ B cells among PerC cells and in blood was not significantly affected by chronic mucosal inflammation **(S1B Fig)**, indicating that those alterations of intestinal B cell subsets by local inflammation were gut mucosal-specific phenomena. Taken together, chronic mucosal inflammation persistently decreased the frequency of regulatory CD5^+^ B cells.

## Discussion

In this study, we investigated the involvement of intestinal CD5^+^ B cells in normal and inflamed colon tissues. Acute mucosal inflammation transiently decreased the frequency of intestinal CD5^+^ B cells, while chronic inflammation persistently reduced the percentage of intestinal CD5^+^ B cells, leading to a CD5^-^ B cell-dominant mucosal condition. Persistent alteration of the intestinal immune cell population by chronic inflammation may be associated with abnormal immune response to resident bacteria in IBD patients.

Recent studies have suggested that B cells play a regulatory role in intestinal inflammation and mucosal homeostasis.[12,15–18] However, not all B cells have a regulatory function in colitis, and anti-inflammatory responses by regulatory B cells are dependent on the quality and quantity of other types of immune cells. For example, *Il10*^*-/-*^ B cells and *Myd88*^*-/-*^ B cells lack an anti-inflammatory property,[16,19] and IL-10-producing regulatory B cells require IL-27 signaling-mediated IL-10-secretion by T cells to confer protection against T cell-mediated colitis.[16] Therefore, classification of the mucosal B cell population and evaluation of its functional aspects in regard to the interaction with other types of immune cells are important to understand the functions of the mucosal immune system in IBD.

CD5 localized on the surface of B cells has been reported to be an important cell surface marker able to distinguish regulatory B cells in infectious and autoimmune diseases.[2,3,20,21] However, this B cell subset also appears to be involved in the pathogenesis of IBD.[12,18] Interestingly, the frequency of peripheral and mucosal CD5^+^ B cells was found to be lower in human UC patients as compared to healthy volunteers,[22,23] and corticosteroid therapy further decreased the percentage of CD5^+^ B cells in UC patients.[22] In the present study, we found that colonic LP CD5^+^ B cells were primarily composed of B220^hi^CD5^lo^, while PerC CD5^+^ B cells possessed mainly the B220^mid^CD5^mid^ (B-1) phenotype, which was previously reported to regulate chronic colitis by generating natural antibodies in response to microbial flora.[10] These results imply that colonic LP CD5^+^ B cells have a phenotype different from that of PerC CD5^+^ B cells and may regulate mucosal inflammation by another mechanism. We also found that colonic LP CD5^+^ B cells express a relatively higher level of TLR9 and produce a greater amount of IL-10 than CD5^-^ B cells with TLR stimulation, especially CpG-DNA. TLR9 seems to play a protective role in patients with IBD [24,25] and TLR9 mRNA levels were reported to be significantly higher in inflamed mucosa from UC patients as compared with normal subjects.[26] Moreover, the expression level of TLR9 in peripheral B cells from IBD patients was found to be greatly increased.[27] Together, these findings suggest that IL-10 secretion via TLR9 signaling may be a potential mechanism used by intestinal CD5^***+***^ B cells for regulating gut inflammation, though additional functional examinations of IL-10-dependent and -independent regulatory mechanisms are required.

Recent studies have identified specific CD5^***+***^ B cell subsets that produce higher levels of IL-10 and regulate inflammation as compared to other B cells. B10 cells characterized as CD5^+^CD1d^high^B cells have been shown to produce abundant IL-10 and ameliorate EAE and DSS-induced colitis,[12,28] while CD5^+^CD24^high^CD38^high^ B cells have been reported to attenuate anti-neutrophil cytoplasmic autoantibody (ANCA)-associated vasculitis by IL-10-production.[29] However, despite multiple mechanistic studies of the CD5-signaling pathway in B cells,[30] it remains unclear whether CD5 is induced by activation or arises from separate precursors through distinctive development. Moreover, the distribution and migration systems of this subset in the human body are largely unexplained.

On the other hand, CD5^***-***^ conventional B cells have been shown to have a pro-inflammatory role in adaptive immune responses via the production of autoantibodies and activation of effecter T cells.[1,31] Those findings suggest that a sustained CD5^-^ B cell-dominant mucosal condition caused by chronic colitis may potentially aggravate disease activity and be involved in the pathogenesis of refractory IBD.

Additionally, it is also important to consider clinical implications in regard to recovery of the frequency of intestinal CD5^+^ B cells following the transient decrease caused by acute inflammation noted in this study. Findings from another study[32] as well as ours obtained by B220-immunohistochemistry examinations of colon specimens (data not shown) indicate that the total number of colonic B cells is profoundly increased and reaches maximum at 14 days after acute DSS treatment, with the population of CD5^-^ B cells showing a greater increase than that of CD5^+^ B cells. After day 14, the total number of B cells then gradually decreases in parallel with recovery of B cell polarization. These results indicate that CD5^-^ B cells are more sensitive to inflammation, while the absolute number of CD5^+^ B cells is more slightly affected by colitis despite their decreased frequency. Moreover, previous studies revealed that the chemokine CXCL13 and its receptor CXCR5 play essential roles in B cell migration.[33–35] Our study found that mucosal inflammation greatly increased the expression of CXCR5 in intestinal CD5^-^ but not CD5^+^ B cells (data not shown), suggesting that CD5^-^ B cells may have a greater attraction to and higher level of infiltration in inflamed mucosa in a CXCR5-dependent manner.

Nevertheless, in addition to our interesting observations, mechanistic and functional studies of mucosal CD5^+^ and CD5^-^ B cells will be required to firmly establish the roles of intestinal B cell subsets in mucosal inflammation and homeostasis. For example, examining the inflammation-mediated phenotypic transformation of CD5^+^ to CD5^-^ on mucosal B cells would be important. In addition, in vivo adaptive transfer experiments to compare intestinal IL-10^+/+^ to IL-10^-/-^ CD5^+^ B cells or IL-10 blocking assays would be useful to address the existence of an IL-10-dependent or -independent regulatory mechanism, while investigation of TLR expression in CD5^+^ and CD5^-^ B cells from chronic inflamed intestinal tissues, or functional analysis of TLRs^-/-^ CD5^+^ B cells would also be of great interest.

In summary, gut inflammation polarizes intestinal B cell subsets by decreasing the frequency of CD5^+^ B cells and inducing a sustained CD5^-^ B cell-dominant condition. To the best of our knowledge, the present results are the first to show that chronic intestinal inflammation sustains a CD5^-^ B cell-dominant condition, which may contribute to the pathogenesis of refractory IBD. Additional functional studies of the roles of intestinal CD5^+^ and CD5^-^ B cells in human IBD will add to our findings.

## Supporting Information

S1 Fig Chronic intestinal inflammation persistently decreases frequency of mucosal CD5^+^ B cells. **(A)** Frequency of CD5^+^ B cells among peritoneal cavity (PerC) cells during acute colitis. **(B)** Flow cytometry was used to determine the frequency of CD5^+^ B cells in PerC and peripheral blood samples during chronic colitis. The experiments were repeated in triplicate. N = 6 at each time point. Data are presented as the mean ± SD. *p<0.05.
(TIF)
