# Supplementary figures and images for: Decreased Frequency of Intestinal Regulatory CD5+ B Cells in Colonic Inflammation
Source: PLoS One. 2016 Jan 4;11(1):e0146191. doi: 10.1371/journal.pone.0146191 (PMC4705109; doi:10.1371/journal.pone.0146191)

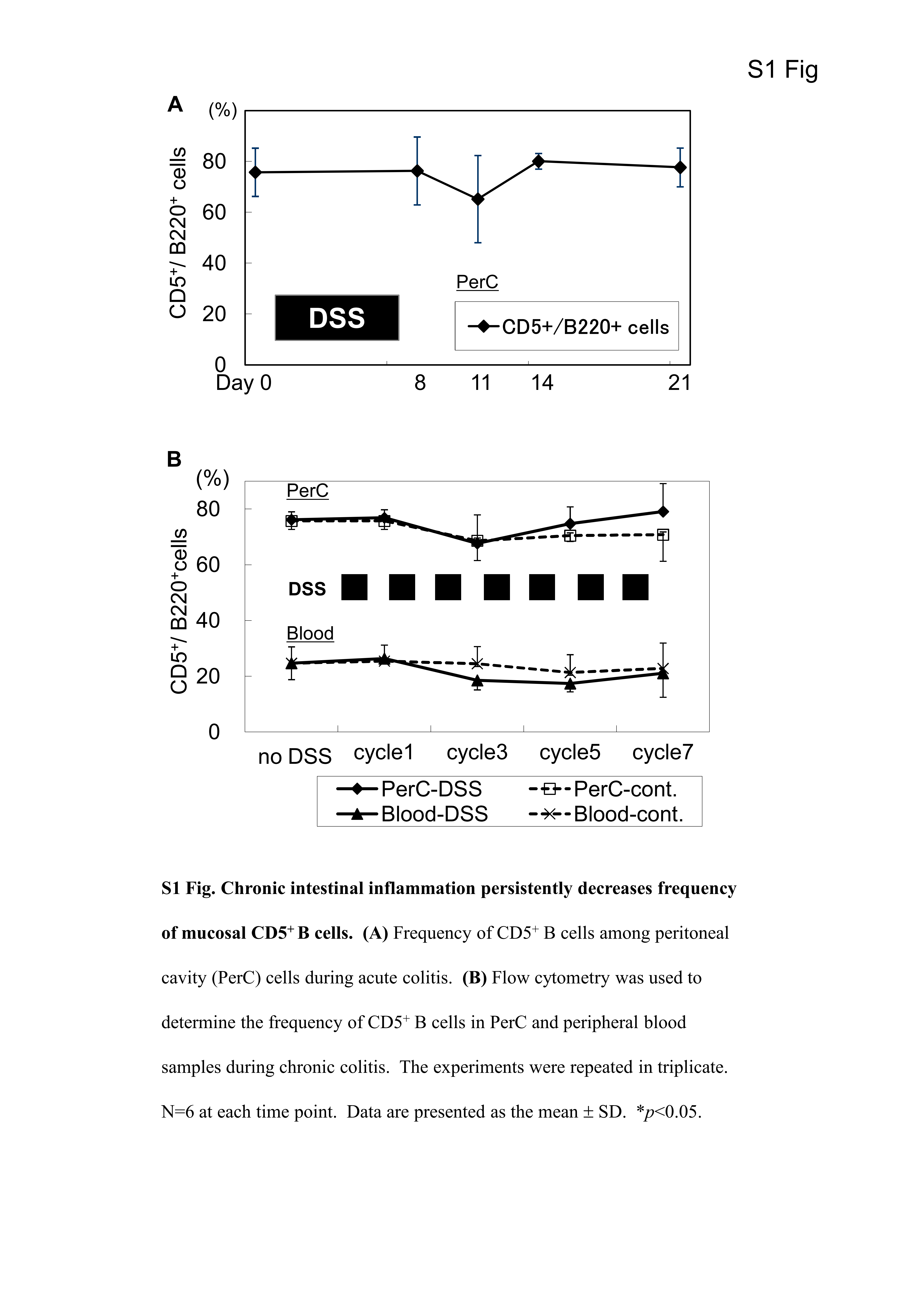

Supplement: S1 Fig — (A) Frequency of CD5+ B cells among peritoneal cavity (PerC) cells during acute colitis. (B) Flow cytometry was used to determine the frequency of CD5+ B cells in PerC and peripheral blood samples during chronic colitis. The experiments were repeated in triplicate. N = 6 at each time point. Data are presented as the mean ± SD. *p<0.05. (TIF) [file pone.0146191.s001.tif]
